# Cognitive basis for the development of aesthetic preference: Findings from symmetry preference

**DOI:** 10.1371/journal.pone.0239973

**Published:** 2020-10-12

**Authors:** Yi Huang, Jinyun Lyu, Xiaodi Xue, Kaiping Peng

**Affiliations:** 1 School of Business and Management, Shanghai International Studies University, Shanghai, PR China; 2 Department of Psychology, Tsinghua University, Beijing, PR China; 3 Tsinghua Laboratory of Brain and Intelligence, Beijing, PR China; University of Glasgow, UNITED KINGDOM

## Abstract

Where is the visual aesthetic preference rooted from and what’s its relationship with the perceptual preference that is emerging early? Do young children naturally prefer some visual stimuli or do they learn to appreciate visual stimuli for aesthetic pleasure? Here, for young preschool children who are on the age that the preferences are developing, we provide findings from a study to show that the interplay between early emerging perceptual sensitivity and perceptual exposure promotes the emergence of preschool children’s aesthetic preferences for simple visual patterns. Specifically in the experiments, 4-year-old children were exposed to either symmetric or asymmetric non-figurative forms in a perceptually demanding game; the group of children who received exposure to symmetric patterns showed aesthetic preference to the exposed patterns, while no preference was found in the group that received exposure to asymmetric patterns. The following recognition test then showed that the symmetric objects were differentiated better and remembered more clearly by the children, indicating that the symmetry was perceptually encoded better. These findings suggest that the early emerging perceptual sensitivity to ‘good features’ such as symmetry provides the prior cognitive prerequisites, allowing visual perceptual exposure to nourish the eventual formation of aesthetic preference. Thus, the preferences for aesthetic appreciation are likely the outcome of the interplay between biological and ecological adaptation.

## Introduction

Visual aesthetic preference refers to people selectively preferring certain visual stimuli that cause them to feel aesthetic pleasure [1]. For example, people tend to find symmetric human faces more attractive than less symmetric ones [2,3]. Such pleasure could occur to people without being explicitly aware of their sources or without clearly associating them to a specific positive value [4,5]; in other cases, people find themselves becoming particularly attracted to an object at first glance, such as an abstract painting in a gallery. Although the visual aesthetic preferences exist across human’s history and different cultures [6,7], and affect people’s daily appreciation of pleasure, their choices of purchase [8] and even the development of their inter-person relationship [9,10], and furthermore, influence many professionals, such as graphic designer [11], artist [12,13] and even marketing strategy maker [14–16], the origin and mechanism of forming these aesthetic preferences are not well understood.

The objectivist view from aesthetic theory, dating back at least to Plato, “saw beauty as a property of an object that produces a pleasurable experience in any suitable perceiver” [17], emphasizing the role of graphic structure or of a feature itself in inducing aesthetic experience. Empirical studies with this view indeed had found human have aesthetic preferences towards certain visual “good features”, such as symmetry [18,19], curved shapes [7,20], moderate complexity [21] and high figure-ground contrast [22]. These features are either explained as being perceived less harmful and thus elicit positive, pleasurable feelings [20,23,24]; or having an organization of perceived “goodness”, meaning they possess a lesser amount of information [25,26] and are easier to perceive, therefore, the higher cognitive processing fluency causes positive aesthetic evaluations [27,28].

Among such features, symmetry is the one been studied extensively that imbedded in different objects from human faces and bodies [2,3,29], to concrete artworks and abstract visual patterns [30,31]. Beyond its link with the attractiveness of human faces, symmetry has been manifested as the best predictor of adults’ aesthetic preference for abstract patterns [32–35]. On the other hand, the perceptual preferences to “good feature” of symmetry have been manifested both for adults and children. For example, adults detect symmetric visual displays faster and more accurately than asymmetric ones [36,37], and remember symmetric patterns better [38,39]. Preschool children pay more spontaneous attention to a symmetric visual pattern over an asymmetric one [40]. Moreover, the perceptual sensitivity to symmetry has been found even emerging from as early as four months old [41]. According to perceptual processing theory, as long as the aesthetic evaluation is not associated with other sources, the experience of perceptual fluency will directly promote the a positive affective feeling [27,42]. Thus, the general aesthetic preference to symmetry seems naturally rooted from the perceptual preference to symmetry, just as it was mentioned by Weyl in his book, “beauty is naturally bonded with symmetry” [43].

However, a recent developmental study on 4-year-old children, on the contrary, had found the aesthetic preference for symmetry dissociates from early emerging perceptual sensitivity to symmetry [40]. With eye-tracking approach in this study, it was found that although preschool children paid more spontaneous attention to a symmetric visual pattern over an asymmetric one, they, unlike adults, did not report any preference when asked to indicate which pattern was more beautiful or preferred. Thus, the perceptual preference and aesthetic preference to “good feature” seem not naturally synchronized. Other recent studies even reported that the aesthetic preference to symmetry over asymmetry can be reversed due to long-term art-training expertise [44]; and that compared to explicit pleasure, the symmetry-induced implicit positive affect was found to be inconsistent across different task settings [45]. Therefore, these findings indicated that the formation of an aesthetic preference to symmetry may also be associated with perceiver’s interactive experience with the objects, as well as the evaluation contexts.

Indeed, beyond the objectivist view in aesthetic theory, researchers with a constructivist view emphasize the interactive experience between subjects and objects in the process of aesthetic preference formation [46,47]. Such research supports the claim that people prefer visual objects that have been exposed to them beforehand, with exposure potentially increasing the familiarity, which suggests that the objects are less harmful and are likely to elevate the feeling of pleasure [27,48]. The phenomenon of repeated exposure increasing people’s aesthetic evaluation is called the mere exposure effect [48–50]. According to this phenomenon, in the development of young children’s aesthetic preference, the large amount of visual experience they acquire every day should affect the formation of their aesthetic appreciation. However, it seems difficult to explain why most people eventually prefer certain kinds of stimuli, such as symmetry, and not their counterparts, such as asymmetry, because, theoretically people should have an equal chance of being exposed to all qualities of stimuli in their daily life. Would the processing fluency also benefit the exposure effect with respect to the formation of aesthetic preference?

Here in this study, we propose a possible mechanism to explain how the perceptual preference supports the development of aesthetic preference. We proposed the early emerging perceptual selectivity towards ‘good features’, e.g., symmetry, provides a perceptual prerequisite for developing children to be more sensitive to visual exposure of the features, causing later encounters with external exposures to selectively promote and shape the aesthetic preference towards those perceptually advantageous features. We focused on 4-year-old children, because in this age, children are already able to express preferences, but their preferences are still in developing, therefore, providing a natural time window to study the process of preference formation. To test the proposed hypothesis, across three experiments, we designed a picture-matching game to have children receive pure perceptual exposure before testing their preferences towards either symmetric or asymmetric pictures. We expected that through the proposed mechanism, visual exposure should affect the preference for symmetry but not asymmetry. After the preference test, we also implemented a recognition task by asking the children to recognize and differentiate a series of pictures they had or had not seen in the game. If symmetry indeed has a perceptual advantage, as reported by previous studies [38,39], symmetric pictures should be remembered and recognized better.

## Experiment 1

### Participants

Sixty-eight 4-year-old children (*M* = 4.45, *SD* = 0.63; 50% female) were recruited from kindergartens. They were randomly assigned to either an exposure to symmetry or an exposure to asymmetry group. Four children were excluded from the final data analysis because of missing data. Each condition included 32 participants (exposure to symmetry: *M* = 4.57, *SD* = 0.60; 50% female; exposure to asymmetry: *M* = 4.34, *SD* = 0.66; 50% female). The sample size was pre-determined by setting the statistic power as 0.95 and the effect size as 0.6 for one-sample t-test, and was calculated in the software of G*Power 3.1.9.2. All written consents were obtained from the parents before the experiments.

### Stimuli and procedures

#### Exposure and preference task

**Fig 1:**
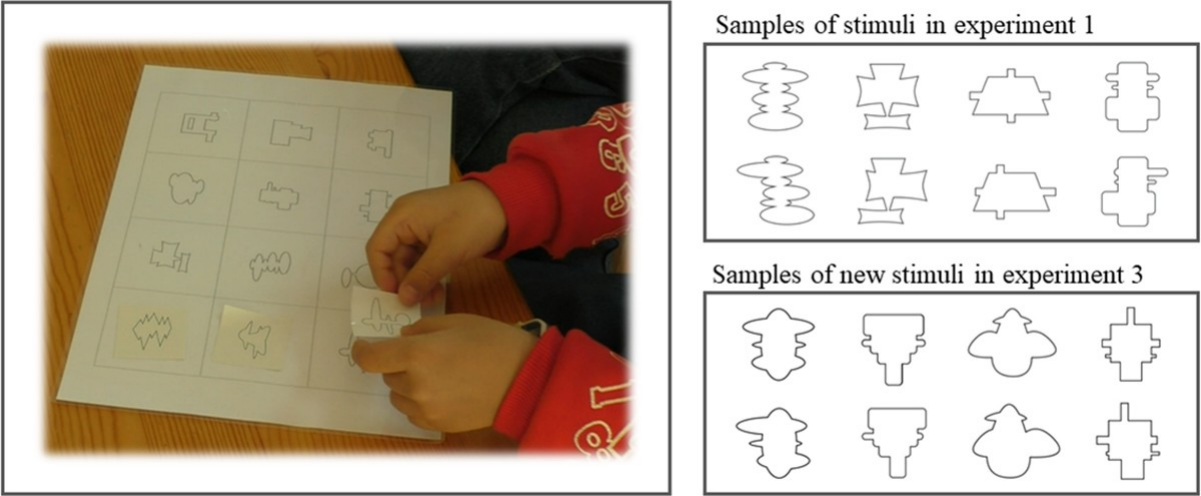
The sticker matching game used to give children perceptual exposure and the stimulus samples of the paired non-figurative forms.

Pairs of pictures of non-figurative forms were created as experimental stimuli. Each pair contained two pictures that showed objects with similar elements (e.g., straight lines, curved lines, rectangles, ovals) that were taken from Photoshop software but with one having a symmetric overall structure and the other asymmetric. Each asymmetric picture was formed by slightly moving the shapes from each symmetric picture either horizontally or vertically (Fig 1), so that with the exception of structural difference (symmetry or asymmetry), the other figural properties (e.g., the degree of curvature) within each picture pair were balanced as much as possible. These pairs were used both in the preference task and separated into individual pictures for the exposure game. To ensure that the children received adequate perceptual exposure and delivered equal amount of attention to symmetric and asymmetric pictures in the exposure game, a sticker matching game was designed. In this game, each participant received a pile of randomly mixed stickers, each having a single form that matched one of the 12 different sample forms on a sticker map. Children were instructed to match each sticker with its corresponding place on the map. The map was 21 cm×29.7 cm and had 12 grids printed on it. The grid size was 6 cm×6 cm, and in the center of each grid, one randomly selected sample form was shown. Each child would play only on the map containing either all symmetric samples or asymmetric samples to obtain exposure to symmetry or asymmetry (Fig 1). Children were told that each sticker would be scored as correct if put onto the right grid. To successfully complete the game, children had to observe and differentiate each picture carefully, which guaranteed perceptual exposure in both conditions.

After completing the sticker game, participants took a short rest by moving around freely in the experiment room for a few minutes before being instructed to do the following preference task. For each trial, children would see one pair of forms presented on an iPad Mini screen. Each pair contained one form they had seen in the sticker matching game, symmetric or asymmetric, and a counterpart, asymmetric or symmetric, they had not seen. Children were instructed to play a role of a judge to point out which form in each pair was more beautiful and they liked better. All 12 pairs were shown in random order, and the location of the symmetric or asymmetric form was counterbalanced on the left or right side of the screen.

#### Recognition test

After completing the preference task, the children took a short break by moving around freely in the room before beginning the recognition test. In this test, the non-figurative forms that the children had played with in the sticker game were presented on an iPad Mini screen one by one in a random order. In addition to the 12 pictures children had seen before, 4 new pictures that were created under the same principles were mixed with the ones that appeared in the exposure paradigm. For each presented picture, children were asked if they had seen or played with this picture before, and their “yes” or “no” answers were recorded by the experimenter.

All the experimental protocols and participant consent were approved and performed under the regulation of Institutional Review Board (IRB) for human research in Department of Psychology in Tsinghua University.

### Data analysis

For the preference task, the percentage of choice of symmetric forms for the symmetry group and the percentage of choice of asymmetric forms for the asymmetry group were calculated as the after exposure preference, and then each was compared with a 50% probability with a one-sample t-test to determine whether the exposure effect exists.

For the recognition task, the correct recognition rate for the pictures the children had seen in the sticker game, and the correct rejection rate for the pictures the children had not seen in the game, were calculated and compared with a 50% probability with a one-sample t-test to test whether the pictures were correctly perceived.

The independent-sample t-test was used when comparing the group differences.

The effect size for all tests across the tasks was Cohen’s *d*, which was calculated as *d* = $\frac{{\bar{X}}_{1}-{\bar{X}}_{2}}{SD}$. And all the comparisons were two-tailed in order to test all the possible differences with a stricter statistical than one-tailed tests can offer.

### Results

**Fig 2:**
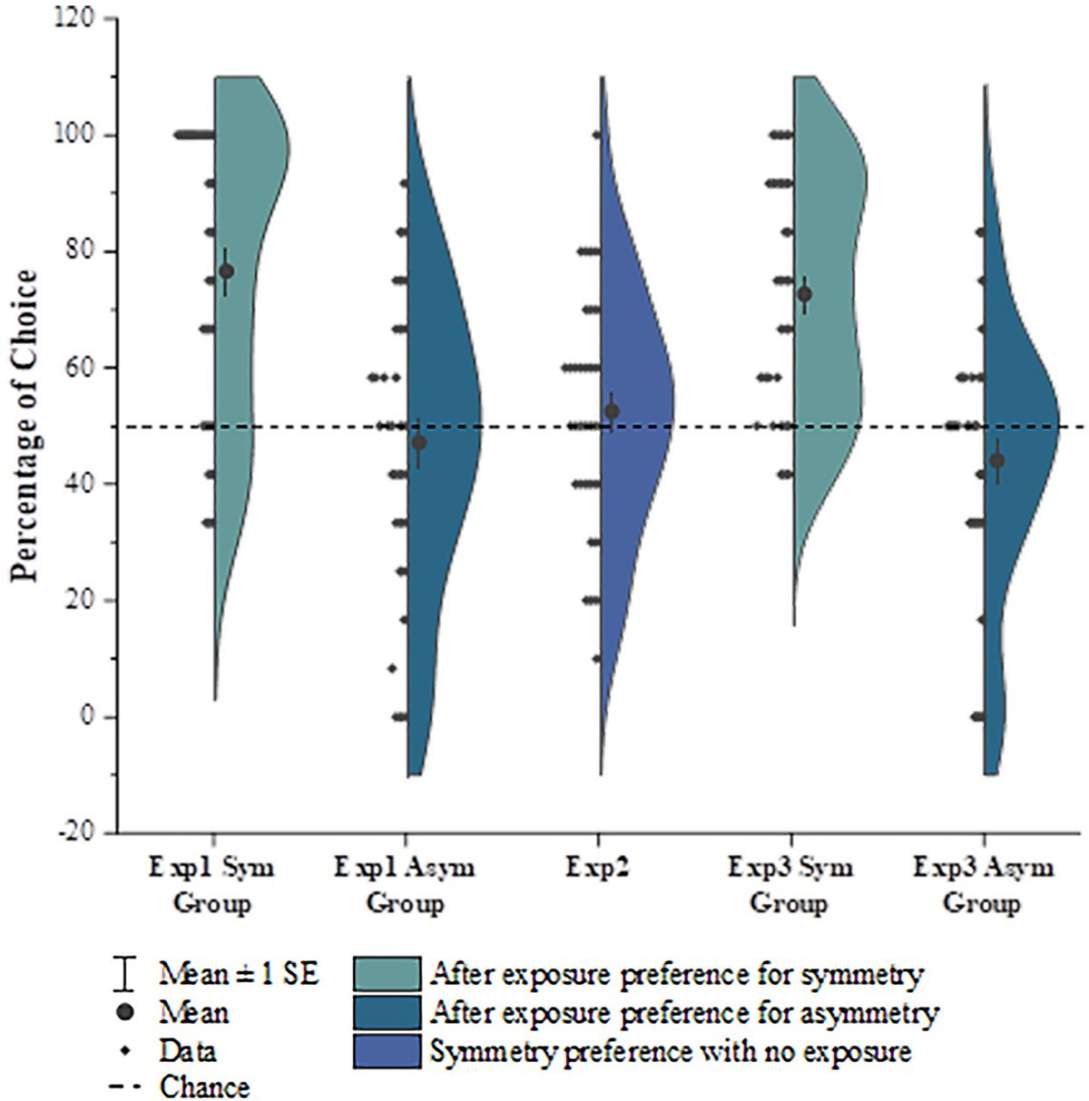
Children’s aesthetic preference across all experiments. Half violin plots with data points on the other half show a kernel density estimate of the full distributions of the percentage of choices for the preferred forms. Both in experiment 1 and 3, children who were exposed to symmetric forms showed a significant preference for symmetry compared with chance, while children who were exposed to asymmetric forms showed no preference. Children with no exposure to any type of form in experiment 2, showed no significant preference.

All children in experiment 1 were able to complete the sticker matching game within 5 minutes (*M* = 198.21 s, *SD* = 62.20 s) and have all the forms matched with the corresponding samples on the map. No group difference was found for the time duration of game completion (*t* (61) = -0.322, *p* = 0.748, *d* = 0.081, *95%CI* [-36.90,26.66]; the completion time of one child in symmetry group was not recorded, so 63 children were included in this test). For the preference task, after the perceptual exposure introduced by the game, the group exposed to symmetry showed significant preference towards symmetric pictures (*M* = 76.56%, *t* (31) = 6.077, *p* < 0.001, *d* = 1.074, *95%CI* [17.65,35.48]), but the group exposed to asymmetry did not show any preference (*M* = 47.14%, *t* (31) = -0.648, *p* = 0.522, *d* = 0.115, *95%CI* [-11.88,6.15]) (Fig 2), thereby supporting our hypothesis that visual exposure would have differing effects regarding perceptually distinct features in influencing children’s aesthetic preference. Next, we further asked whether the symmetric forms we used in the experiment indeed had a perceptual advantage that was in line with previous studies using different formats of symmetry stimuli (e.g., dot patterns) [40].

**Fig 3:**
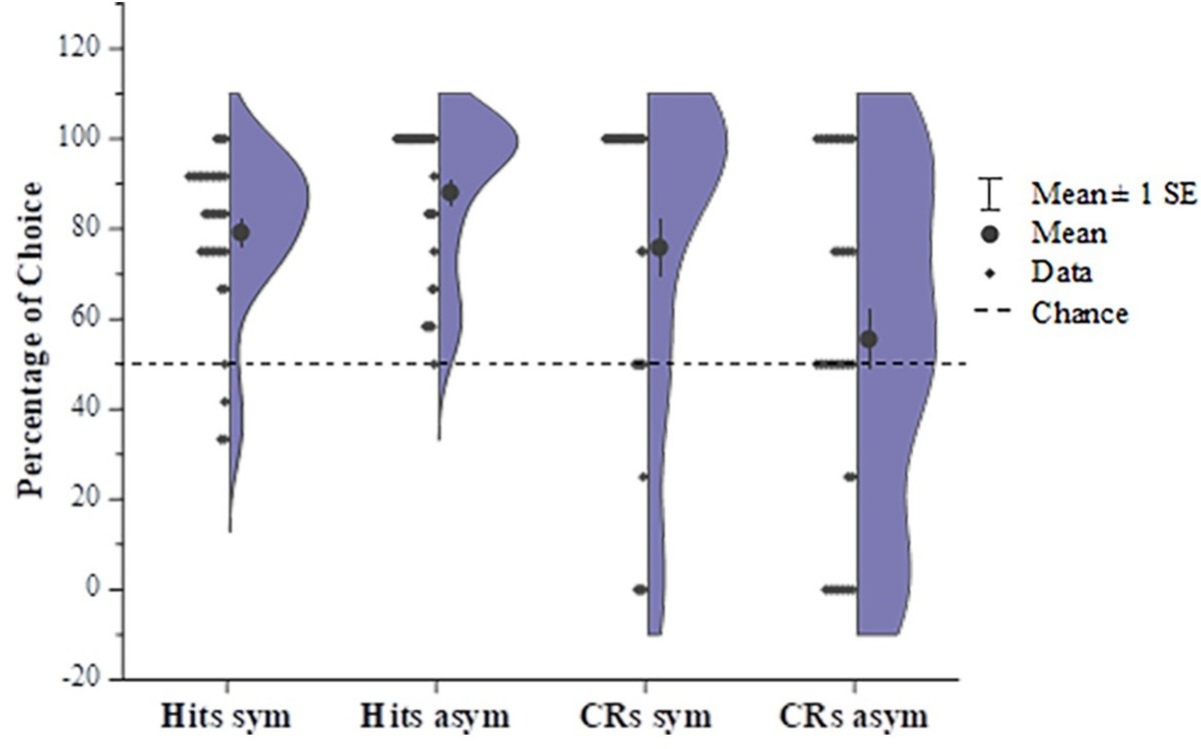
Correct recognition and rejection rates in the recognition test for experiment 1. Half violin plots with data points on the other half show a kernel density estimate of the full distributions of the percentage of choices. Children who played with symmetric pictures had a significant correct rejection rate for the pictures that did not appear in the game, while children who played with asymmetric pictures could not correctly reject the pictures that they had not seen in the game compared with chance. Both groups of children correctly recognized the pictures they had played in the sticker game.

We tried to answer this question by determining whether the symmetric pictures were encoded more precisely by children in the following recognition test. The two groups of children, both the one given exposure to symmetry and the one given exposure to asymmetry, significantly recognized the pictures they had played with compared to a 50% probability of chance(symmetry: *M* = 79.17%, *t* (31) = 9.251, *p* < 0.001, *d* = 1.635, *95%CI* [22.74,35.60]; asymmetry: *M* = 88.02%, *t* (31) = 12.708, *p* < 0.001, *d* = 2.246, *95%CI* [31.92,44.12]) (Fig 3), which reflected a successful exposure induced by the game. The group difference showed marginal significance (*t* (62) = -2.037, *p* = 0.046, *d* = 0.509, *95%CI* [-17.54,-16.55]). However, only the group in the exposure to symmetry condition made significantly correct rejections of the additional unseen pictures (symmetry: *M* = 75.78%, *t* (31) = 4.030, *p* < 0.001, *d* = 0.712, *95%CI* [12.73,38.83]; asymmetry: *M* = 55.47%, *t* (31) = 0.827, *p* = 0.415, *d* = 0.146, *95%CI* [-8.02,18.96]), and the correct rejection rate of the exposure to symmetry group was significantly higher than that of exposure to asymmetry group (*t* (62) = 2.207, *p* = 0.031, *d* = 0.552, *95%CI* [1.92,38.71]) (Fig 3), suggesting a more precise encoding regarding symmetry during testing with the unseen pictures as distractors.

## Experiment 2

In experiment 1, we found that no preference emerged from the exposure to the asymmetry group for neither asymmetric nor symmetric forms. This is in line with the previous finding that children at this age have not shown aesthetic preference for symmetry-related features [40]. However, we were still unsure if, with no exposure, children’s aesthetic preference for symmetry already existed for the current line-drawing forms and if the current results were caused by it. To rule out this possibility, we recruited another participant group in experiment 2 and tried to replicate the finding with the presently implemented forms to demonstrate that 4-year-olds do not yet show spontaneous aesthetic preference for symmetry.

### Participants

Thirty-two 4-year-old children (*M* = 4.47, *SD* = 0.24; 50% female) were recruited in another kindergarten to participate in experiment 2. The sample size was pre-determined by setting the statistic power as 0.95 and the effect size as 0.6 for one-sample t-test, and was calculated in the software of G*Power 3.1.9.2. All written consents were obtained from the parents before the experiments.

### Stimuli and procedures

The same picture preference task in experiment 1 but without the beforehand sticker exposure was conducted with a laptop in a different kindergarten in experiment 2; and due to the limited testing time this kindergarten allocated for the children to participate in the experiments, the number of trials was reduced to ten. For each trial of the experiment, the sizes of the pair of pictures were 180×180 pixels on the laptop screen with a resolution of 1440×900 pixels. Across the trials, the locations of the symmetric or asymmetric forms were counterbalanced on the left or right side of the screen, and the participants sat approximately 50 cm away from the screen. As within the experiment 1, the participants were instructed by the experimenter to point out which form in each pair was more beautiful or they liked better without receiving any exposure to the forms in advance.

All the experimental protocols and participant consent were approved and performed under the regulation of Institutional Review Board (IRB) for human research in Department of Psychology in Tsinghua University.

### Results

We calculated their percentage of choice for symmetric forms and compared them with 50% probability by a one-sample t-test. Similar to the previous study using dot patterns to represent symmetry/asymmetry [40], we did not find children to have a significant choice of preference for the symmetric non-figurative forms either (*M* = 52.50%, *t* (31) = 0.680, *p* = 0.501, *d* = 0.120, *95%CI* [-5.00,10.00]) (Fig 2), which ruled out the hypothesis of a preexisting preference for symmetry without exposure.

## Experiment 3

Thus far, two experiments showed that visual exposure to symmetry promoted young children’s aesthetic symmetry preference while visual exposure to asymmetry had no similar effect and that the symmetric pictures were perceptually encoded better by children. Although the finding supported our hypothesis on the mechanism for development of aesthetic preference—that a perceptual encoding advantage made symmetric structure more sensitive to visual exposure that helped children at a young age with building an aesthetic preference towards it—one issue was still not clear: whether the aesthetic symmetry preference was specifically built to the identical stimuli that the children had been exposed to in the game or was established for extended symmetric stimuli that even never been played with by the children. To clarify this issue, we further performed experiment 3 by rerunning the exposure game, preference task, and recognition test with the same experimental procedures but different stimuli settings on a new group of children. In the new setting, children saw different pictures of stimuli between the exposure game and preference task; and the number of pictures they had seen in the exposure game and the number of pictures as distractors that they had never seen was identical for the recognition test.

### Participants

Sixty-four 4-year-old children (*M* = 4.04, *SD* = 0.32; 50% female), who had never participated in specialized art training, were recruited from different kindergartens and randomly assigned to either the exposure to symmetry or the exposure to asymmetry group (exposure to symmetry: *M* = 4.07, *SD* = 0.29; 53% female; exposure to asymmetry: *M* = 4.00, *SD* = 0.35; 47% female). The sample size was pre-determined by setting the statistic power as 0.95 and the effect size as 0.6 for one-sample t-test, and was calculated in the software of G*Power 3.1.9.2. All written consents were obtained from the parents before the experiments.

### Stimuli and procedures

In experiment 3, the exposure game, preference task, and recognition test like in experiment 1 were rerun on a new group of participants with the same procedures but different stimulus settings, in which children saw different pictures in the exposure game and the preference task, and they were provided an equal number of pictures they had played with and pictures they had not seen in recognition task. More specifically, in addition to the original 12 pairs of the stimuli, 24 more pairs were created for experiment 3 using the same principles. The participants were exposed to 12 forms (symmetry or asymmetry) randomly selected from the entire 36 form pairs and performed a preference task with 12 different pairs of forms. Then, the children finally performed the recognition test on a mixture of the 12 pictures of forms they had seen in the exposure sticker game and 12 pictures of forms of the same type (symmetry or asymmetry) they had never seen.

All the experimental protocols and participant consent were approved and performed under the regulation of Institutional Review Board (IRB) for human research in Department of Psychology in Tsinghua University.

### Data analysis

For the preference task, the percentage of choice of symmetric forms for the symmetry group and the percentage of choice of asymmetric forms for the asymmetry group were calculated as the after exposure preference, and then each was compared with a 50% probability with a one-sample t-test to determine whether the exposure effect exists.

For the recognition task, the correct recognition rate for the pictures the children had seen in the sticker game, and the correct rejection rate for the pictures the children had not seen in the game, were calculated and compared with a 50% probability with a one-sample t-test in order to determine whether the pictures were correctly perceived. Since the number of the new pictures was equivalent to the number of the old ones, the approach of signal detection theory approach was adopted, and the d’ for each group was calculated as d’ = z (Hits)–z (False Alarms).

The independent-sample t-test was used when comparing the group differences.

The effect size for all tests across the tasks was Cohen’s *d*, which was calculated as *d* = $\frac{{\bar{X}}_{1}-{\bar{X}}_{2}}{SD}$. And all the comparisons were two-tailed in order to test all the possible differences with a stricter statistical power than one-tailed tests can offer.

### Results

The results for the preference task in experiment 3 showed that children who received symmetry exposure in the sticker game also showed significant symmetry aesthetic preference in the preference choice task even for the new symmetric pictures they had never played with in the game (*M* = 72.66%, *t* (31) = 6.374, *p* < 0.001, *d* = 1.13, *95%CI* [15.41,29.91]), while the children who received asymmetry exposure still showed no preference either for the new symmetry pictures or the new asymmetry pictures (*M* = 44.01%, *t* (31) = 1.525, *p* = 0.137, *d* = 0.27, *95%CI* [-14.00,2.02]) (Fig 2). These results suggest that this exclusive exposure effect on building a symmetry aesthetic preference not only exists for familiar symmetric stimuli but could also be extended to other unexposed symmetric patterns.

**Fig 4:**
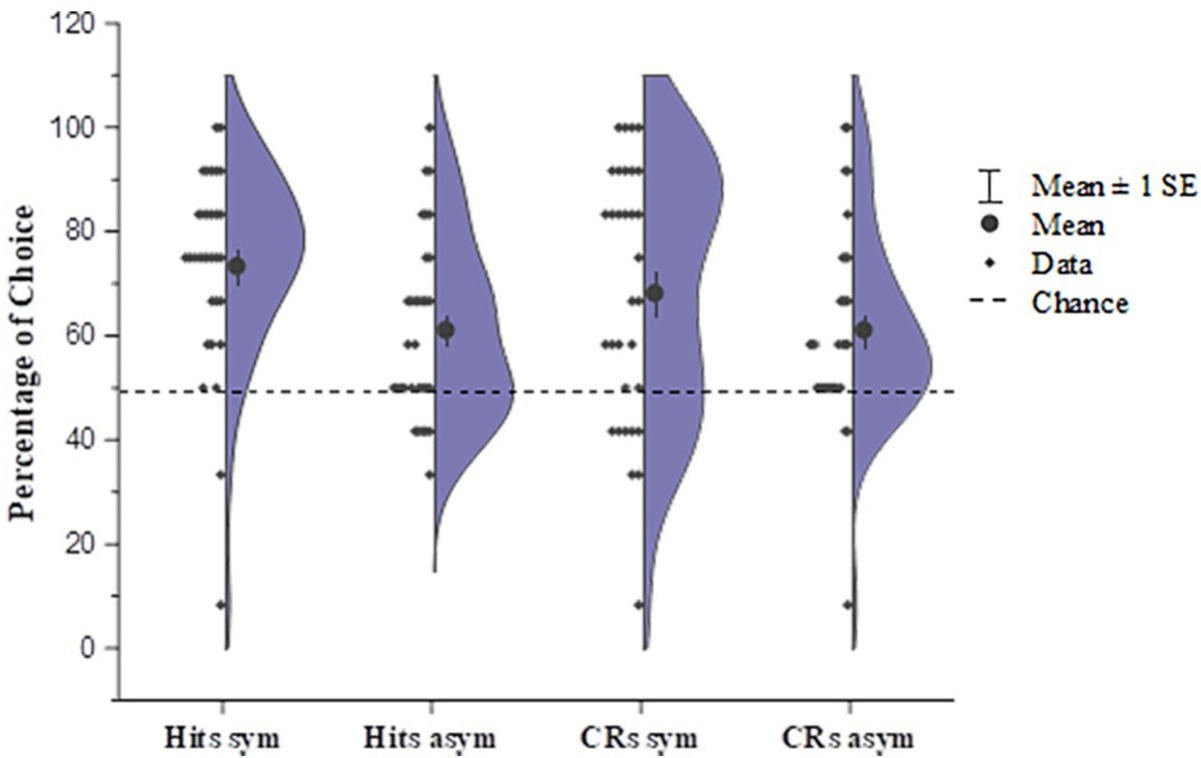
Correct recognition and rejection rate in the recognition test for experiment 3. Half violin plots with data points on the other half show a kernel density estimate of the full distributions of the percentage of choices. Both groups of children correctly recognized the pictures they had seen in the sticker game and rejected the pictures they had not seen in the game. Children who played with symmetric pictures had higher correct recognition rates compared with children who played with asymmetric pictures.

**Fig 5:**
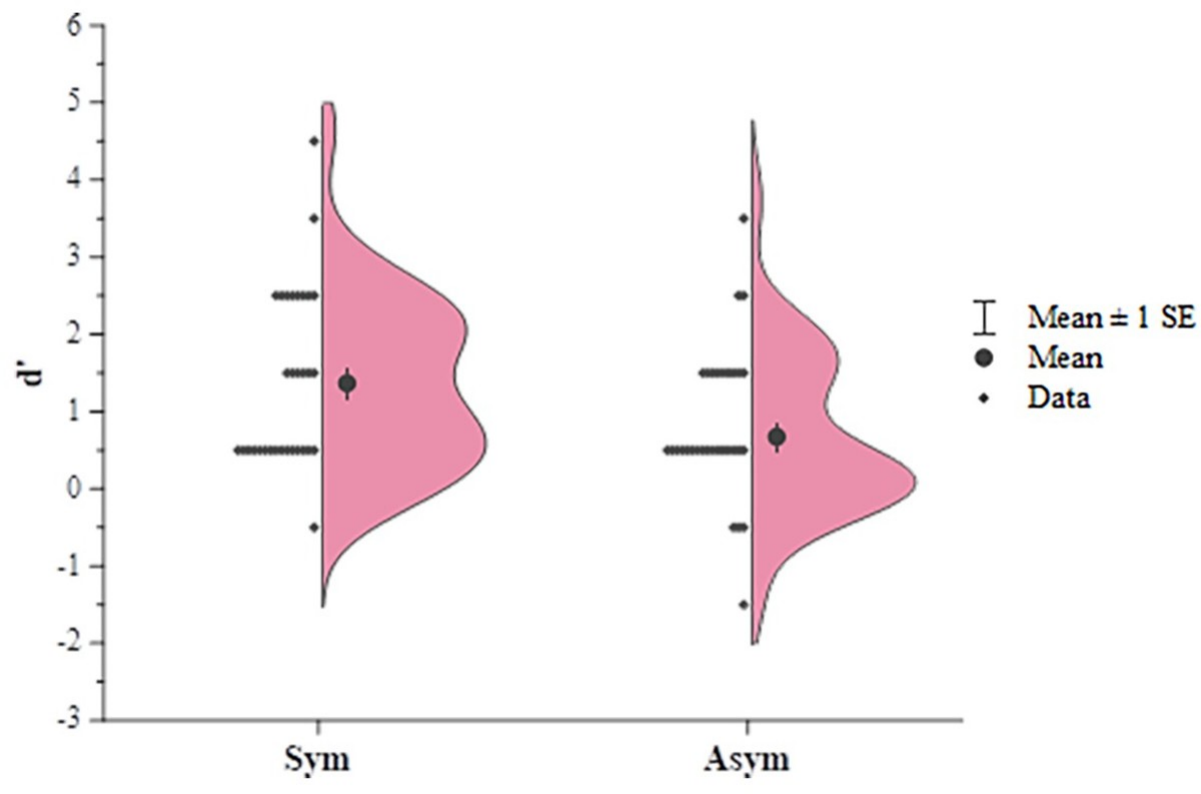
The d’ value calculated using signal detection analysis for the recognition test in experiment 3. Half violin plots with data points on the other half show a kernel density estimate of the full distributions of d’ values. Children who had played with symmetric pictures had higher d’ values in the picture detection task than children who had played with asymmetric pictures, indicating higher perceptual encoding efficiency for symmetric forms.

For the following recognition test, similar to the results in the experiment 1, both groups of children could recognize the pictures they had played with (symmetry: *M* = 73.44%, *t* (31) = 6.899, *p* < 0.001, *d* = 1.22, *95%CI* [16.51,30.37]; asymmetry: *M* = 61.20%, *t* (31) = 3.667, *p* = 0.001, *d* = 0.648, *95%CI* [5.00,17.43]), and they could also make correct judgements about the pictures they had not seen in exposure phase (symmetry: *M* = 68.23%, *t* (31) = 4.186, *p* < 0.001, *d* = 0.740, *95%CI* [9.35,27.11]; asymmetry: *M* = 61.20%, *t* (31) = 3.375, *p* = 0.002, *d* = 0.600, *95%CI* [4.43,17.96]) (Fig 4). These results indicated a successful exposure induced by the exposure game. The group difference in the recognition rates was significant (*t* (62) = 2.679, *p* = 0.009, *d* = 0.670, *95%CI* [3.11,21.37]), which indicates a more precise encoding with respect to symmetric forms; although the group difference on the correct rejection rates was not shown (*t* (62) = 1.284, *p* = 0.204, *d* = 0.321, *95%CI* [3.91,17..97]), and the possible reasons for which are detailed in the Discussion section. Next, we further performed signal detection analysis to compare the perceptual encoding efficiency of the two groups, and we found that the d’ of the exposure to symmetry group was significantly higher than that of the exposure to asymmetry group (*t* (31) = 2.579, *p* = 0.012, *d* = 0.672, *95%CI* [0.16, 1.23]) (Fig 5), which indicated young children’s better encoding efficiency for symmetric structures over asymmetric structures.

## Discussion

Though the appreciation of aesthetic preferences play an important role in people’s development of positive mental life, in building their lifelong interests, and with its origin as a focus of discussion for a long time, the mechanism for developing aesthetic appreciation preference is still unclear. To our knowledge, the current study has for the first time revealed the interplay between perceptual advantage and visual exposure in contributing to the development of young children’s aesthetic preference. We chose to focus on aesthetic preference for symmetry in current experiments for two reasons. First, symmetry is regarded as the core feature mediating aesthetic appreciation [1,12,27,51]. Second, the previous finding that 4-year-olds did not show aesthetic preference for symmetry but had perceptual preference for symmetry [40] provided a natural time window to study how aesthetic preference emerges and its relation with perceptual preference. We replicated the finding that 4-year-olds did not spontaneously choose the symmetric objects as more pleasurable; interestingly, however, after receiving pure perceptual exposure by playing the picture matching game, the preference for symmetry emerged. Meanwhile, the exposure did not cause any effect on the similar but counterpart asymmetric pictures. Furthermore, this effect not only existed for the pictures exposed to children but also extended to new pictures with similar symmetric structures that the children had never seen. The subsequent recognition test showed that children indeed differentiated the symmetric forms better, which suggests that the symmetry was better perceptually encoded. Therefore, as we hypothesized, the “good feature” accepted and “defined” early by the perceptual system provided an advantageous cognitive foundation for the preference of aesthetic appreciation to be built upon; then, the preferences were finally promoted by the nourishment of an external exposure experience. The aesthetic preference, thus, might be essentially the outcome of both biological and ecological adaptation.

The current findings increased our understanding of the cognitive mechanism underlying the development of aesthetic appreciation. Cognitively, two factors have been proposed for causing aesthetic pleasure, perceptual familiarity and processing fluency [27]. Zajonc suggested that familiar objects evoke positive emotions because of the implication that the object is unlikely to be harmful [50,52]. According to the theory of processing fluency, aesthetic pleasure is a function that increases with how easily the object has been processed [27]. However, neither of the factors can solely explain the current findings. First, although both symmetric and asymmetric pictures were familiar to the children after the exposure, as both the exposed pictures were remembered well, only the symmetric pictures were aesthetically preferred. Thus, familiarity alone seems inadequate to promote children’s aesthetic appreciation. Second, although our study did not directly test processing fluency for the symmetric and asymmetric stimuli, the recognition test showed that the symmetric pictures were indeed encoded better, which is usually linked with high processing fluency. Therefore, if higher processing fluency can simply raise the aesthetic preference for children, we should expect, even with no exposure, that children would prefer symmetric pictures; however, spontaneous preference was not shown in either the current or the previous study. Hence, at least for 4-year-olds’ aesthetic appreciation, according to current data, both familiarity and processing fluency in the cognitive process might be necessary. This may be because one single factor is not salient enough for young children’s less mature evaluation systems [53], but future studies are needed to verify this.

Our findings also provide new insight into understanding the relationship between the early emerging perceptual preference and the later developed subjective preference with value evaluation. The visual preferences that start as early as infancy have been used to infer the development of children’s cognitive process or value evaluation, such as for moral judgement [54], social evaluation [55] or logical induction [56]. However, the early emerging preferences focused on by these previous studies were usually induced by a certain task linked with a specific domain. Therefore, those preferences were basically a rudimentary mode for the full-fledged capacities. The current study demonstrates that even pure perceptual preference, which does not contain any rudimentary evaluation element, could be the prerequisite for the development of high-level subjective value.

Another interesting implication came from the findings of the experiment 3, in which we found the exclusive exposure effect in promoting a symmetry aesthetic preference can be transferred to similar symmetric structures that children had not observed at all in the exposure phase. This implies that at the age of 4, the formation of aesthetic preference might not only be restricted to specific stimuli they are familiar with but could also happen in the general conceptual level. A previous study found that 3- to 4-year-old children are already able to complete a transfer learning task when they learn to use tools and its relevant implicit rule [57]; our results further suggest that concept learning should also play an important role in children’s formation of subjective aesthetic preference. It would be interesting for future studies to further test this transferring for many different types of stimuli; for example, if children get exposure to symmetric single objects, would the promoted aesthetic symmetry preference be transferred to symmetric dot patterns?

Our game-driven paradigm of exposure is explicit and without the participants’ foreknowledge of the following choice of preference task. Though this setting should have good ecological validity, the exposure children encounter in their daily life could had occurred in many other ways before the experiment, implicitly or repeatedly. Previous studies on the mere exposure effect have found that different manipulations of the exposure, such as subliminally or with multiple repetitions, would affect the outcomes caused by the exposure. For example, too many instances of exposure have been reported to decrease aesthetic evaluation [58]. Consequently, how different settings of exposure interact with the cognitive preferences and affect the development of children’s aesthetic appreciation could be further explored.

Some of the current study’s limitations need to be further addressed in future research. First, although the perceptual sensitivity to symmetry showed an overall advantage over asymmetry in the recognition task (Figs 3–5), which were in line with previous studies [38,39], some parts of the results were not fully replicated between experiments 1 and 3. Specifically, in experiment 1, the correct rejection rate for unexposed pictures in the exposure-to-symmetry group was significantly higher than that of the exposure-to-asymmetry group, but it was not replicated in experiment 3; while in experiment 3, the correct recognition rate for exposed pictures in the exposure-to-symmetry group was significantly higher than that of the exposure-to-asymmetry group, which was not shown in experiment 1; and the correct recognition rate of the asymmetry group even show marginally higher trend than that of the symmetry group in experiment 1, but not in experiment 3. One possible reason that accounts for these instabilities might be that the recognition task was the last one that was performed, which would have been at a time when the 4-year-old children might already have become relatively tried and less able to concentrate their attention, after having completed two other tasks. In addition, the lower number of trials used to accommodate participants who are young children might decrease the statistic stability as well. Second, although we encouraged the children to point out the more beautiful picture in each pair, we also promoted their action by asking which one they liked better, especially when they hesitated. For adults, the “beauty” and “liking” were not always necessarily synchronized [59], but we are unsure whether children on the age of four are able to separate these two processes, between which future study could try to differentiate. Third, although we attempted to match the figural properties (e.g. curvature, contrast) beyond symmetry as much as possible for each picture pair, it is possible that the within-pair differences pertaining to other dimensions, such as complexity, which was recently reported to be negatively associated with the picture’s degree of symmetry [60], are still unavoidable. In the current study, it is less likely that the complexity played a major role in the aesthetic evaluation process, because no any preference to asymmetry that should be considered more complex to be preferred [61] was found; however, future developmental studies that are primarily focused on other figural features’ association with aesthetic evaluation would better help to illustrate these issues.
